# Testing the Arabic-Saudi Arabia version of the Rome IV Diagnostic Questionnaire for functional gastrointestinal disorders for Children living in Saudi Arabia

**DOI:** 10.3389/fped.2022.1055513

**Published:** 2023-01-25

**Authors:** Mai A. Khatib, Elham A. Aljaaly

**Affiliations:** Clinical Nutrition Department, Faculty of Applied Medical Sciences, King Abdulaziz University, Jeddah, Saudi Arabia

**Keywords:** functional gastrointestinal disorders, Rome IV diagnostic criteria, children in Saudi Arabia, functional constipation, functional dyspepsia, Rome IV-SA

## Abstract

Functional gastrointestinal disorders (FGID) are a worldwide phenomenon described by painful, recurrent or chronic gastrointestinal (GI) symptoms. Variable types of FGID exist in a significant portion of children in Saudi Arabia (SA). While the studies and reports on child FGID are limited, the available ones show a notable significance of FGID in children in SA. The self-report Rome IV Diagnostic Questionnaire (DQ) globally recognizes the selection of symptom criteria and incidence thresholds. Using such a questionnaire would help clinicians provide a provisional diagnosis, serve as a case definition for epidemiological surveys, and identify inclusion criteria for clinical trials. This research aimed to pilot test the collective FGIDs prevalence among preschool children in Jeddah city and its countryside of Saudi Arabia, using Rome IV DQ in Arabic-SA. Of the 59 responses, 11.8% (*n* = 7), 5% (*n* = 3), 1 (1.6%), and 1 (1.6%) participants have functional dyspepsia, functional constipation, functional irritable bowel syndrome, and functional aerophagia, respectively according to the Rome IV criteria. The tested translated DQ in this study was the first translated version available in Arabic- SA, which could provide researchers and clinicians in SA with a diagnostic tool for FGIDs. However, because this study is a pilot study in a new field, the conclusions cannot be extrapolated to the demographic of the targeted population of children. The same researchers plan a larger study to use the current results and a larger calculated sample to assess FGIDs prevalence in children 4+ years old in Jeddah and its countryside, Saudi Arabia.

## 1. Introduction

Functional gastrointestinal disorders (FGID) are a worldwide phenomenon described by painful, recurrent or chronic gastrointestinal (GI) symptoms (1–3). FGID includes esophageal, gastroduodenal, enteric conditions, abdominal pain syndrome, gallbladder and sphincter, and anorectal conditions that are functional. It also includes disorders: newborns and toddlers and functional disorders: children and adolescents (4–6). Structural or biochemical abnormalities do not explain the symptoms of FGID (6) and are not identified by organic, systemic or metabolic diseases that justify the existing symptoms (7, 8). Functional stomach discomfort, defecation issues, and functional nausea and vomiting are a few of the most common symptoms in children (9). These abnormalities often manifest in combination (2).

Child FGID prevalence rates in Saudi Arabia (SA) differ according to the type of disorder. In a 2014 meta-analysis and comprehensive review of the published research, the prevalence of irritable bowel syndrome (IBS) in Asian children (including Saudi children) was estimated to be 2.8%–25.7% (10). In 2016, a study stated a 32.2% prevalence rate of constipation in Saudi children (11), and the frequency of diarrhea among children below the age of six reached 7.5% (12). Although in 1995, 4,290 patients underwent endoscopic examinations for seven years (1995–2001), it was then seen that there was a frequency of 2.6 out of 1,000 cyclic-vomiting syndrome cases (13). Moreover, a panel in 2015 found that 32% of mothers had to change their infants’ formula due to infantile colic (14). This indicates that various types of FGID exist in many children in SA. While the studies and reports on child FGID are limited, the available ones show a notable significance of FGID in children in SA. Risk factors for FGID, such as constipation, include family history (11).

The cornerstone for diagnosing FGIDs is established as reported gastrointestinal (GI) symptoms, such as diarrhea and abdominal pain. The Rome IV Diagnostic Questionnaire (DQ), a widely used self-reported DQ, allows the choice of the symptom criteria and occurrence levels (15). Using such a questionnaire would help clinicians provide a provisional diagnosis, serve as a case definition for epidemiological surveys, and identify inclusion criteria for clinical trials (15). The collective incidence of FGIDs in preschool children in Jeddah city and its countryside of Saudi Arabia “to the best of the researchers’ knowledge” have not been previously evaluated. To measure the prevalence of FGIDs among children in Saudi Arabia needs, a validated tool such as the ROME IV Diagnostic (DG) Questionnaire for FGIDs. Arabic is the official language in Saudi Arabia Kingdom, which is part of the Middle East countries and the Arab league. Therefore, the study adapted the ROME IV DG Questionnaire, which is in English and translated it into Arabic-SA to be completed by parents of participants who prefer to respond in Arabic. The translation process was based on RF guidelines (16).

Pilot studies represent vital phases of the research process, including examining research tools (17). Therefore, the objectives of the current pilot study were as follows:

1. To assess and refine additional developed questions by the researchers to define possible risk factors related to the prevalence of FGIDs.
2. To test and redraft the survey questions for the translated Rome IV DQ FGIDs for Children aged four and above and the constructed researchers' questions, as well as the research protocol before conducting further extensive studies involving the assessment of FGIDs prevalence among children living in Saudi Arabia.
3. To identify if factors include a family history of GI disorders, food consumption, early determinants of gut microbiota, type of maternal delivery and early life feeding, and the use of herbal and relaxation remedies as possible risk factors for the common FGIDs among Saudi children.

### 1.1. Outcome measures

The primary measure is to calculate the overall incidence number of existing FGIDs cases for the period the translated Rome IV DQ for Children aged four and above was examined. The secondary measures are to examine associations of family history of GI disorders, life events (type of maternal delivery and early life feeding and everyday food consumption), and the use of traditional pharmacologic treatment (herbal and relaxation remedies) with the prevalence of FGIDs cases.

## 2. Material and methods

### 2.1. Method

#### 2.1.1. Study design

This preliminary cross-sectional study was designed to examine the reliability and validity of two parts questionnaires. (1) developed for general information about children, and (2) a translated into Arabic-SA version tool of the Rome IV DQ FGIDs for Children. The designed study tests the two parts of the questionnaire to define participants, assess the incidence of FGIDs cases in preschool children in Jeddah, Saudi Arabia and possible pre-defined risk factors.

The study’s conduct and data collection were during the COVID-19 pandemic when access to kindergartens in Jeddah and the conduct of in-person interviews were minimal. Therefore, the authors of the present work hosted the questionnaire on Google Forms and distributed the link through kindergarten schools in Jeddah city, targeting parents of children four years old and above. To ensure the completeness of survey items and to reduce missing responses, the authors applied mandatory responses to all survey questions by allowing navigation through the instrument using the “Next button” only after answering the question. Respondents were also allowed to navigate the survey questions using the Back buttons to allow them to review and change their answers whenever applied. In addition to the required responses to the survey questions, another optional button was available to authorize respondents to provide comments about the questionnaire for the authors' consideration.

### 2.2. Material

#### 2.2.1. Setting, sample selection and participants

##### 2.2.1.1. Settings

Governmental-sponsored kindergarten in Jeddah city, Saudi Arabia

##### 2.2.1.2. Study size:

Calculation of Sample Size

According to the latest figure provided by the Ministry of Education in the academic year 2019–2020, approximately 16,000 preschool students were in Jeddah city and its countryside. The number of governmental kindergartens in the Jeddah district and its countryside is 100. The average number of students in each kindergarten is 160, with an average of 25 children in each class. Therefore, the total number of children in the kindergartens in the Jeddah district is about 16,000.

The study used the Cochran approach [*n* = (Z2 PQ)/ e2] to resolve a minimum sample size (18). A 95% confidence interval was used, and the average prevalence of functional constipation (2.07%) in SA is within the level of precision (e) equal to 2%. Therefore, the used Cochran's formula [*n* = (Z2 PQ)/ e2], where Z (the value indicating the confidence level) = 1.96, *p* = 0.0207, Q (standard deviation of the outcome variable) = 1–0.0207 and e (the desired margin of error) = 2% allowed for estimating a sample size for the whole population as *n* = 195 out of 16,000 preschool children.

For this pilot study, about 30% of the sample size = of 19.5 was enough for examining the research tool. However, an extra (20%) was added to cover the effect of non-response. Therefore, 24 parents of students from the governmental-sponsored kindergartens in Jeddah city non-randomly participated in this study to evaluate the practicability and research protocol prior to the performance of the full-scale study.

### 2.3. Participants recruitment

Volunteer parents of children aged 4+ years in Jeddah City were invited *via* schools to respond to surveys concerning their children's possible diagnosis with FGIDs and other related information. The response rate exceeded the targeted number of participants (256%) and a total of 59 parents signed the informed consent forms and responded to the online questionnaire as parents with multiple siblings of one family were invited to fill the questionnaire more than ones based on number of children in the house., Data collection took place from March 2021 to April 2021.

#### 2.3.1. Inclusion and exclusion criteria

##### 2.3.1.1. Inclusion criteria

Parents of preschool children who agreed to participate and self-report the study questionnaire and their children are meeting the following criteria:

- Age: four to six years (boys and girls)
- Enrolled in governmental/private/international kindergartens in Jeddah district in the academic year of 2020–2021.
- Living in Jeddah or its countryside

##### 2.3.1.2. Exclusion criteria

Parents of preschool children who did not agree to participate or their children are:

- Less than four years of age
- Not enrolled in kindergartens in the Jeddah district in the academic year 2020–2021.
- Living outside Jeddah or its countryside

The study tool: An online self-administered questionnaire by preschool children's parents to obtain data for a two parts questionnaire.

1. **First part:** a developed based on the literature questionnaire (11, 19–22) to retrieve general information about the examined population, including children's demographics, previous diagnosis with a gastrointestinal condition, family history of GI disorders, type of maternal delivery and neonatal feeding, commonly consumed foods, and the use of herbal and relaxation remedies as a form of managing to relieve symptoms of GI discomfort.
2. **Second part:** a 60-items translated into Arabic-SA tool of the Rome IV DQ FGIDs for Children aged four and above following the Room Foundation guidelines for tool translation (16). The Rome IV DQ included five sections that aimed to self-reporting any gastrointestinal symptoms concerning the area above the belly button, areas around or below the belly button, bowel movements, nausea and vomiting, and other gaseous symptoms such as burping and swallowing air. This questionnaire has previously been approved and used by many researchers to identify and screen various GI symptoms based on the subjective reporting of patients/parents. The questions are designed to identify specific problems and symptoms related to the digestive tract (specifically: the esophagus, stomach, small intestine, and colon). These symptoms may or may not apply to all children, which could help define the incorporation criteria of the current investigation. The questionnaire was translated by the researchers of this study and made available in the Arabic-SA language. The preliminary results of the translation process included 4-steps, which was guided by the Rome Foundation (RF) have been presented at the 2021 conference for the American Society for Nutrition (ASN). This work was also published (16). As per RF guidelines, a cognitive debriefing process was conducted following the translation process. This was to cross-culturally compare and validate the translated to Arabic-SA questionnaire. A panel of ten members took part in the process from 26 February to 12 March 2021. The process requires the panel's checking of every questionnaire item and receiving their feedback regarding the question's clarity, cultural adaptation, language, and acceptability of the translated question. Subsequently, a sample of five health care professionals who were mothers of +4 years children was selected to “electronically” complete the questionnaire. Participants' feedback on the language and cultural issues was obtained. Following the completion of the cognitive debriefing process, the decision to use the validated tool was made by RF. A scoring algorithm was provided by the Rome Foundation (RF) to be used for research diagnosis purposes, to be able to identify FGIDs prevalence as follows: Functional Abdominal Pain (FAP), functional dyspepsia (FD), Abdominal Migraine (AM), Functional Constipation (FC), Functional Nausea (FN), Irritable Bowel Syndrome (IBS), Non-retentive Fecal Incontinence (RFI), Cyclic Vomiting Syndrome (CVS), Functional Vomiting (FV), Aerophagia (A), and Adolescent Rumination Syndrome (ARS).

### 2.4. Statistical analysis

Frequency and percentages were displayed to identify the socio-demographic variables of children and their general information. Algorithm scoring of specific questions was based on Rome IV foundation guidelines to identify the type of functional gastrointestinal diseases and their prevalence. Participants who were suffering from FGIDs and the participants who were clear from FGIDs were pooled as cases and controls, respectively. The Chi-square test was used to identify the association between these diseases and family history of GI disorders. Multivariate Regression Analysis was conducted to reveal predictors for FGIDs. All figures were presented as raw data. No information was missing since the Google forms program requires all questions to be answered for the questionnaire to proceed. Significance was deemed if the *p*-value was less than 0.05, with the significance level reported as *p ≤ 0.05, **p ≤ 0.01, ***p ≤ 0.001. Statistical Package for the Social Sciences (SPSS 27) was used to examine the data. Graphs were prepared using Graphpad Prism 9.

### 2.5. Ethical approval

This study commenced following reviewing the research protocol by the research team and having ethical approval from the Research Ethics Committee at the Faculty of Applied Medical Science, King Abdulaziz University, with Reference no. FAMS-EC2021-03. The approval of the local School Health and Education Directorate Authorities in the Jeddah district was obtained to access information related to enrolled children in kindergartens in Jeddah city. Consent forms were collected anonymously from all respondents, as parents signed the consent form by answering a mandatory question included in the online questionnaire about the participant's agreement to participate in the study, meaning that answering the questionnaire was dependent on accepting the consent of participating. Moreover, researchers confirmed their volunteering participation before recruitment to the study.3

## 3. Results

### 3.1. Demographics of the sampled schoolchildren

A total of 59 responses were collected. Of them, 61% (*n* = 36) were males and 39% (*n* = 23) were females. 78% (*n* = 46) of the recruited children were Saudi, and 22% (*n* = 13) were non-Saudi. All of the children lived with both of their parents. Other demographics of the study sample are listed in (Table 1).

**Table 1:** Demographic characteristics of the sampled children (*n* = 59).

Variables		Frequency	Percent (%)
What gender is your child?	Male	36	61
	Female	23	39
How old is your child?	Below 4 years	0	0%
	4 years and above	59	100%
What is your child's nationality?	Saudi	46	78%
	Non-Saudi	13	22%
Where does your child live?	Jeddah	49	83%
	countryside of Jeddah	10	17%
Type of delivery of your child?	Normal	43	73%
	C-section	16	27%
When your child was born, he/she was:	A full-term infant	0	0%
	A preterm infant	59	100%
What kind of school does your child go to? (response: *n* = 42)	International	8	14%
	None	5	9%
	Private	32	55%
How many siblings does your child have? (response: *n* = 41)	One	29	49%
	Two	7	12%
	No one	5	8%
Out of his siblings, where does your child rank? (response: *n* = 38)	3rd	7	12%
	Oldest One	28	47%
	Youngest	3	5%
Whom does your child live with?	Both parents	59	100%
	Mother only	0	0%
	Father only	0	0%

### 3.2. Prevalence of FGIDs

Of the 59 responses, 11.8% (*n* = 7) participants have met the criteria of Rome IV for FC, 5% (*n* = 3) participants have met the criteria of Rome IV for FD, 1 (1.6%) participant has met the criteria of Rome IV for IBS, and 1 (1.6%) participant has met the criteria of Rome IV for aerophagia (Table 2).

**Table 2:** Incidence of FGIDs among the recruited pilot data.

Prevalence *of FGIDs* in percentage (n)	Functional Dyspepsia	Irritable Bowel Syndrome	Abdominal Migraine	Functional Abdominal Pain-nos	Functional Constipation	Functional vomiting	Aerophagia
percentage (n)	5% (3)	1.6% (1)	0% (0)	0% (0)	11.8% (7)	0% (0)	1.6% (1)

FGIDs, Functional gastrointestinal disorders.

### 3.3. Family history and previous diagnosis with a gastrointestinal condition

Only 7% (*n* = 4) of the recruited children were previously diagnosed with a gastrointestinal condition. Five (8.5%) children had a family history of dyspepsia/indigestion, 22% (*n* = 13) of the children with a family history of acid reflux, heartburn and GERD, 27% (*n* = 16) of the children with a family history of belching, bloating, and flatulence, 25% (*n* = 15) of the children with a family history of constipation, 12% (*n* = 7) of the children with a family history of diarrhea, and 3% (*n* = 2) of the children with a family history of nausea and vomiting. IBS is prevalent among 24% (*n* = 14) of children's families and peptic ulcer disease is prevalent only among 3% (*n* = 2) (Table 3).

**Table 3:** Family history and previous diagnosis with gastrointestinal condition.

Questions	Answers		
		Yes	No
Has your child been previously diagnosed with any gastrointestinal condition?	Frequency	4	55
	Percentage (%)	7%	93%
**Is there any family history of the following general gastrointestinal problems?**			
Dyspepsia/Indigestion	Frequency	5	54
	Percentage (%)	8.5%	91%
Acid Reflux, Heartburn, GERD	Frequency	13	46
	Percentage (%)	22%	78%
Nausea and Vomiting	Frequency	2	57
	Percentage (%)	3%	97%
Peptic Ulcer Disease	Frequency	3	56
	Percentage (%)	5.1%	94.9%
Abdominal Pain Syndrome	Frequency	1	58
	Percentage (%)	1.7%	98.3%
Belching, Bloating, Flatulence	Frequency	16	43
	Percentage (%)	27%	73%
Biliary Tract Disorders, Gallbladder Disorders and Gallstone Pancreatitis	Frequency	0	59
	Percentage (%)	0%	100%
Constipation	Frequency	15	44
	Percentage (%)	25%	75%
Diarrhea	Frequency	7	52
	Percentage (%)	12%	88%
Crohn's disease	Frequency	0	59
	Percentage (%)	0%	100%
Irritable bowel disease (IBS)	Frequency	14	45
	Percentage (%)	24%	76%

GERD, Gastro-esophageal reflex disease; IBS, Irritable bowel syndrome.

### 3.4. Relationships between the main outcome (prevalence of FGIDs) and possible factors

#### 3.4.1. Relationships of the common prevalent of FGIDs with family history and previous diagnosis with a gastrointestinal condition

**Figure 1:**
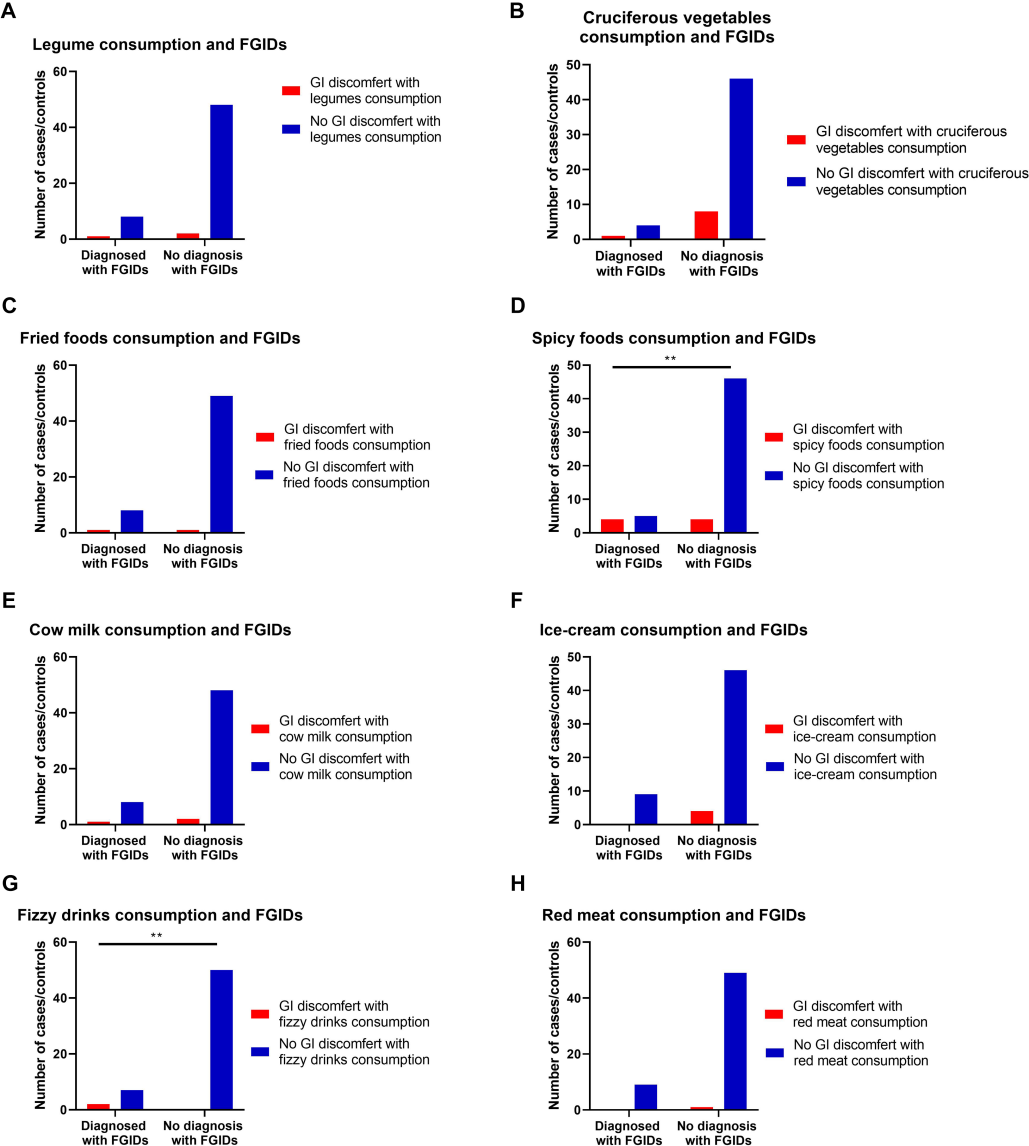
The relationship between the most common FGIDs and family history of gastrointestinal (GI) diseases. Data represent frequency (*n* = 59) of (•) cases and (•) controls of (**A**) functional dyspepsia and family history of peptic ulcers, (**B**) Functional abdominal pain and family history of abdominal pain, (**C**) Functional constipation and family history of constipation, (**D**) functional vomiting and family history of vomiting, (**E**) irritable bowel syndrome and family history of irritable bowel syndrome (IBS), and (**F**) Functional aerophagia and family history of a Aerophagia. Chi-square test was used to test the relative risk of family history of certain diseases and their possible accompanied FGIDs. Data at which values differed significantly, *p ≤ 0.05, **p ≤ 0.01, ***p ≤ 0.001.

Family histories were significantly related to some of the children's prevalent FGIDs (Table 4). For example, it was found that children with a family history of peptic ulcers were more likely to develop functional FGIDs than those with no family history of peptic ulcers (*p* = 0.01) (OR =  14.0, 95% CI = 1.11–175.34). Moreover, it was found that children with a family history of abdominal pain were more likely to develop functional FGIDs than those with no family history of abdominal pain (*p* = 0.01) (OR =  17.82, 95% CI = 0.67–474.65). (Figure 1 and Table 4).

**Table 4:** Relationship between gastrointestinal family history and functional gastrointestinal diseases.

Variables	Cases *N* (%)	Control *N* (%)	*p-*value	RR	95% CI
**Functional gastrointestinal diseases and family history of peptic ulcer**					
Family history of peptic ulcers.	2 (22.2)	1 (2)	0.01**	2.62	0.52–13.04
No family history	7 (77.8)	49 (98)			
**Functional gastrointestinal diseases and family history of GERD**					
Family history of GERD	3 (33.3)	6 (66.7)	0.374	1.13	0.82–1.55
No family history	10 (20)	40 (80)			
**Functional gastrointestinal diseases and family history of vomiting**					
Family history of vomiting	1 (11.1)	1 (2.0)	0.16	1.71	0.42–6.90
No family history	8 (88.9)	49 (98.0)			
**Functional gastrointestinal diseases and family history of constipation**					
Family history of constipation	4 (44.4)	11 (22)	0.155	1.20	0.87–1.66
No family history	5 (55.6)	39 (78)			
**Functional gastrointestinal diseases and family history of IBS**					
Family history of IBS	2 (22.2)	12 (24)	0.908	0.98	0.76–1.26
No family history	7 (77.8)	38 (76)			
**Functional gastrointestinal diseases and family history of abdominal pain**					
Family history of abdominal pain syndrome	1 (11.1)	0 (0)	0.01**	7.25	1.38–110.4
No family history	8 (88.9)	50 (100)			
**Functional gastrointestinal diseases and family history of belching, bloating, and flatulence**					
Family history of belching, bloating, and flatulence	4 (44.4)	12 (24)	0.20	1.17	0.87–1.59
No family history	5 (55.6)	38 (76)			

RR, Relative risk; CI, Confidence interval; GERD, Gastro-esophageal reflex disease; IBS, Irritable bowel syndrome.

Data was analyzed using Chi-square test. Data at which values differed significantly, *p ≤ 0.05, **p ≤ 0.01, ***p ≤ 0.001.

#### 3.4.2. Relationships of the common prevalent FGIDs with the type of maternal delivery

**Figure 2:**
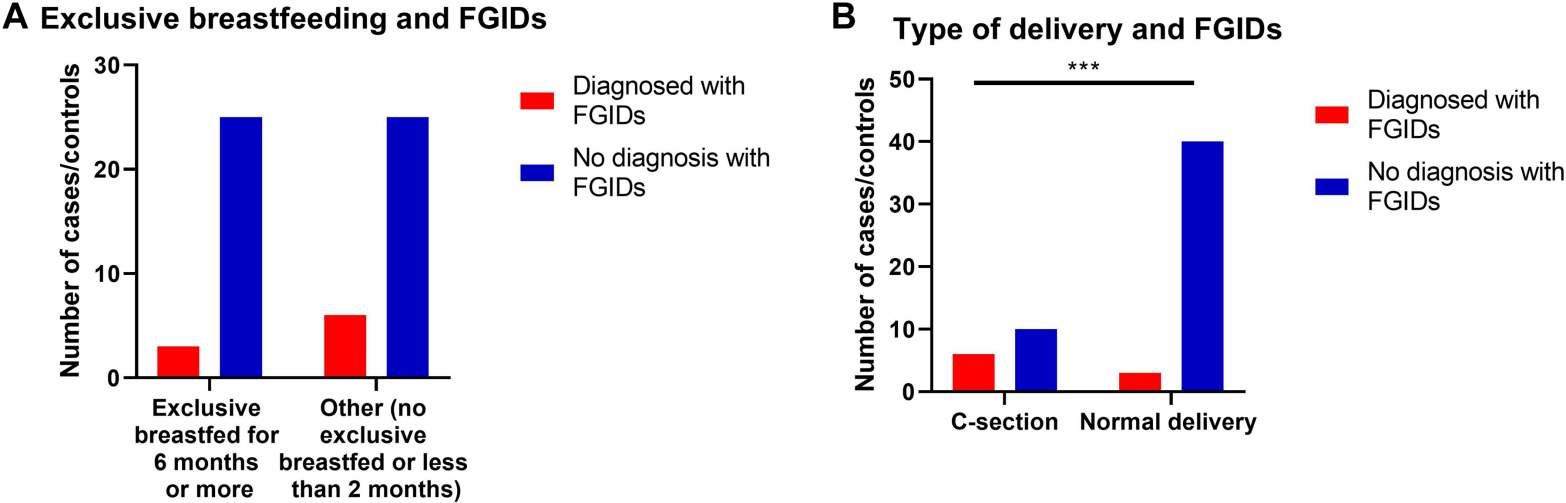
The relationship between FGIDs and type of delivery and neonatal feeding. Data represent frequency (*n* = 59) of (•) cases and (•) controls of (**A**) neonatal feeding and FGIDs, (**B**) type of delivery and FGIDs. Chi-square test was used to test the relative risk of type of delivery and neonatal feeding on developing FGIDs. Data at which values differed significantly, *p ≤ 0.05, **p ≤ 0.01, ***p ≤ 0.001.

Prevalence of FGIDs among children when they were neonates was significantly influenced by the type of delivery, as children born *via* C-Section were significantly more probable to develop FGIDs compared to typically delivered children (*p* = 0.004) (OR =  8.0, 95% CI = 1.69–37.67) (Table 5 and Figure 2).

**Table 5:** Relationship between type of delivery and neonatal feeding and functional gastrointestinal diseases.

Variables	Cases *N* (%)	Control *N* (%)	*p*-value	RR	95% CI
**Type of delivery**					
Normal delivery	3 (33.3)	40 (80)	0.004*	1.48	1.00–2.19
Cesarean section	6 (66.7)	10 (20)			
**Neonatal feeding up to 6 months or more**					
Exclusive breastfeeding	3 (33.3)	24 (48)	0.30	1.12	0.90–1.39
Other	6 (66.7)	26 (52)			

RR, Relative risk; CI, Confidence interval.

Data was analyzed using Chi-square test. Data at which values differed significantly, *p ≤ 0.01.

#### 3.4.3. Relationships of the common prevalent FGIDs with the commonly consumed foods

**Figure 3:**
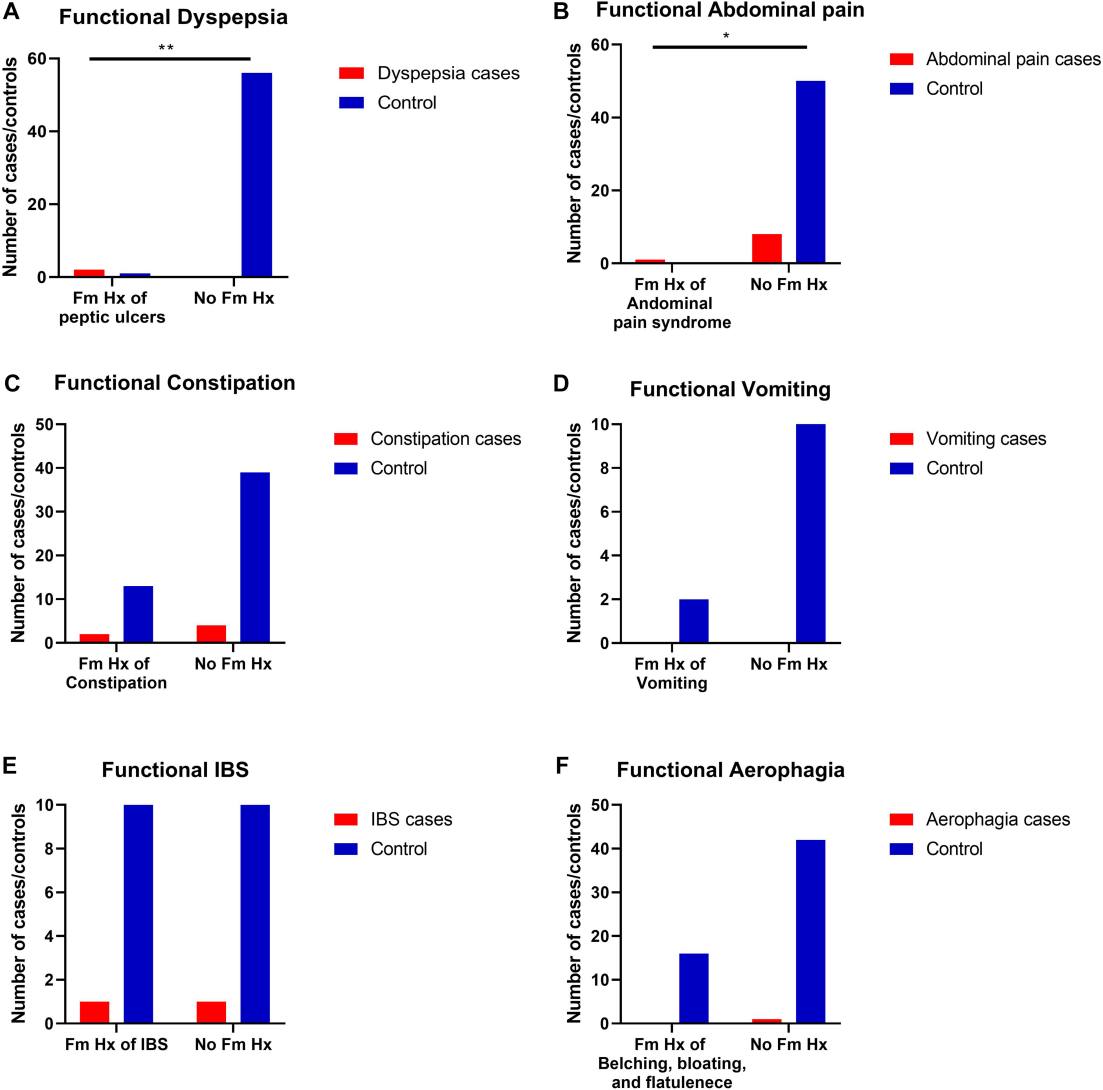
The relationship between FGIDs and some common foods consumption. Data represent frequency (*n* = 59) of (•) cases and (•) controls of (**A**) FGIDs and legume consumption, (**B**) FGIDs and cruciferous vegetables consumption, (**C**) FGIDs and fried foods consumption, (D FGIDs and spicy foods consumption, (**E**) FGIDs and cow milk consumption, (**F**) FGIDs and ice-cream consumption, (**G**) FGIDs and fizzy drinks consumption, and (**H**) FGIDs and red meat consumption. Chi-square test was used to test the relative risk of common food consumption on developing discomfort GI symptoms in cases of children with FGIDs. Data at which values differed significantly, *p ≤ 0.05, **p ≤ 0.01, ***p ≤ 0.001.

Results also show the effect of some commonly consumed foods on developing GI discomfort symptoms in both children's groups (with or without FGIDs). For example, consuming spicy foods and fizzy drinks were found to significantly develop discomfort GI symptoms in cases of children with FGIDs (*p* = 0.003, OR =  9.2, 95% CI = 1.74 to 48.63, and *p* = 0.001, OR =  33.67, 95% CI = 1.47 to 771.43, respectively) (Table 6, Figure 3).

**Table 6:** Relationship between factors affecting functional gastrointestinal diseases and gastrointestinal symptoms.

Variables	Cases *N* (%)	Control *N* (%)	*p*-value	RR	95% CI
**Legume consumption**					
Having GI discomfort when consuming legumes	1 (11.1)	2 (4)	0.37	1.28	0.57–2.88
No GI symptoms	8 (88.9)	48 (96)			
**Cruciferous vegetables consumption**					
Having GI discomfort when consuming cruciferous vegetables	1 (11.1)	4 (8)	0.75	1.06	0.67–1.67
No GI symptoms	8 (88.9)	46 (92)			
**Fried food consumption**					
Having GI discomfort when consuming fried food	1 (11.1)	1 (2)	0.16	1.71	0.42 to 6.90
No GI symptoms	8 (88.9)	49 (98)			
**Spicy food consumption**					
Having GI discomfort when consuming spicy food	4 (44.4)	4 (8)	0.003**	1.80	0.89–3.62
No GI symptoms	5 (55.6)	46 (92)			
**Cow milk consumption**					
Having GI discomfort when consuming cow milk	1 (11.1)	2 (4)	0.37	1.28	0.57–2.88
No GI symptoms	8 (88.9)	48 (96)			
**Ice-cream consumption**					
Having GI discomfort when consuming ice-cream	0 (0.00)	4 (8)	0.37	0.83	0.74–0.94
No GI symptoms	9 (100)	46 (92)			
**Fizzy drinks consumption**					
Having GI discomfort when consuming fizzy drinks	2 (22.2)	0 (0.00)	0.001***	22.22	1.08–100.00
No GI symptoms	7 (77.8)	50 (100)			
**Red meat consumption**					
Having GI discomfort when consuming red meat	0 (0.00)	1 (2)	0.66	0.84	0.75–0.94
No GI symptoms	9 (100)	49 (98)			
**Using herbal medicine to relieve symptoms of GI discomfort**					
Use herbal medicine	2 (22.2)	8 (16)	0.64	1.07	0.77–1.49
Do not use herbal medicine	7 (77.8)	42 (84)			
**Using relaxation exercise (message) to relieve symptoms of GI discomfort**					
Use message	1 (11.1)	6 (12)	0.93	0.98	0.71–1.36
Do not use message	8 (88.9)	44 (88)			

RR, Relative risk; CI, Confidence interval; GI, Gastrointestinal.

Data was analyzed using Chi-square test. Data at which values differed significantly, *p ≤ 0.05, **p ≤ 0.01, ***p ≤ 0.001.

#### 3.4.4. Relationships of the common prevalent of FGIDs with the use of herbal and relaxation remedies

**Figure 4:**
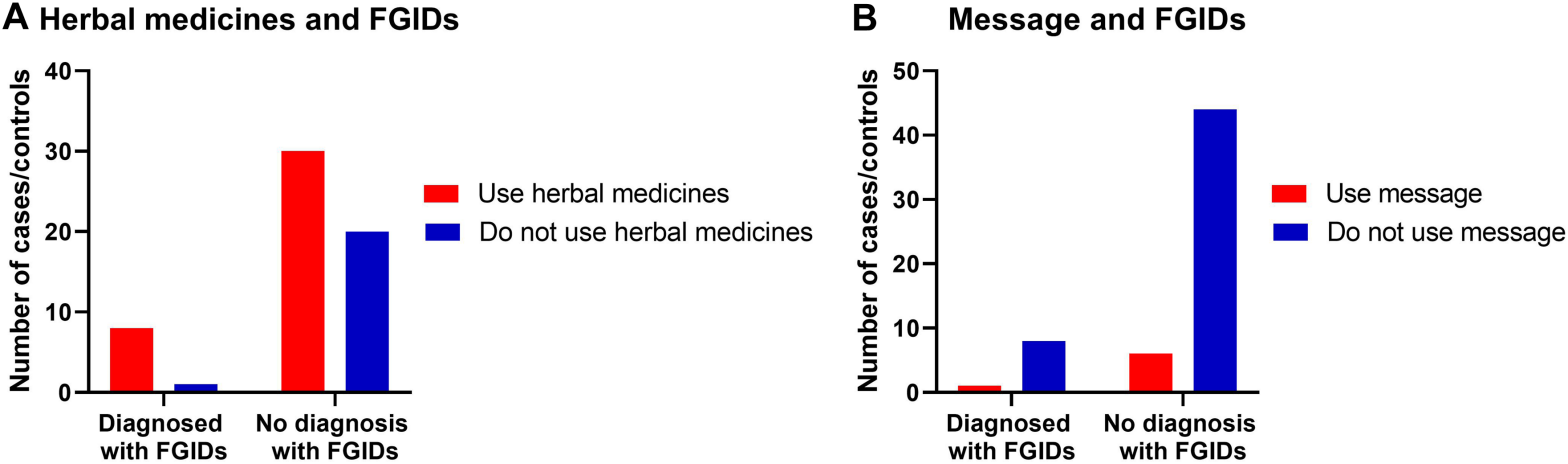
The relationship between FGIDs and using herbal medicine and relaxation therapy. Data represent frequency (*n* = 59) of (•) cases and (•) controls of (**A**) using herbal medicine among children with FGIDs, (**B**) using relaxation therapy among children with FGIDs. Chi-square test was used to test the relative risk of using herbal medicine and relaxation therapy among children with FGIDs. Data at which values differed significantly, *p ≤ 0.05, **p ≤ 0.01, ***p ≤ 0.001.

No statistically significant relationship between herbal medicine use and relaxation exercises in reliving any GI discomfort symptoms in children with FGIDs (Table 6, Figure 4).

#### 3.4.5. Relationships of all independent variables with the prevalence of FGIDs

It was determined by a logistic regression model integrating all independent variables that undergoing a C-section during delivery increased the risk of acquiring FGIDs (*p* = 0.01, Odds ratio of 7.88, CI of 1.65–37.67) (Table 7).

**Table 7:** Multivariate regression analysis: tests of FGIDs predictors among family histories of diseases and neonatal delivery and feeding.

Variables	*p*-value	OR	95% CI
**Type of delivery**			
Cesarean section	0.01*	7.88	1.65–37.67
**Neonatal feeding up to 6 months or more**			
formula or less than 2 months breastfeeding	0.36	1.03	0.95–1.12
**Gastrointestinal family history**			
Family history of dyspepsia	0.97	6312.20	6.7E−2–5.9E + 2
Family history of Acid Reflux, Heartburn, GERD	0.98	146.17	5.2E−1–4.0E + 1
Family history of Nausea and Vomiting	0.98	0.01	2.7E−1–1.0E + 1
Family history of Peptic Ulcers	0.98	0.001	0.00- 0.01
Family history of Constipation	0.97	43.15	1.0E−1–1.7E + 1
Family history of IBS	0.98	2.22	1.0E−4–4.9E + 4
Family history of Belching, Bloating, Flatulence	0.97	0.10	2.1E−71–5.6E + 6
Family history of Abdominal Pain	0.01*	0.12	0.120.129

OR, Odds ratio; CI, Confidence interval; GERD, Gastro-esophageal reflex disease; IBS, Irritable bowel syndrome; GERD, Gastro-esophageal reflex disease.

Data was analyzed using multivariate regression analysis. Data at which values differed significantly, *p ≤ 0.01.

## 4. Disscusion

As far as the authors are aware, this study is the first to shed light on the FGIDs incidence cases among preschoolers in Jeddah city and to look at possible related risk factors using a self-administered approach for a Rome IV FGIDs criteria (Arabic-SA version) in addition to other questions to define the targeted children for FGIDs diagnosis.

Utilizing the recent version of the Rome criteria to identify FGIDs incidence in children in Saudi Arabia was reported in a limited number of published studies (11, 22). However, these studies were limited to some of the Rome foundations FGIDs criteria. Of them, Hasosah et al. (11) and Khayat et al. (22) have found that 32.2% and 13.5% of their study sample of children, respectively, had functional constipation (FC), while the highest prevalence of FGIDs in the current findings was for FC (11.8%). This result supports the commonality of FC in Saudi Arabia. It is well known that people with FC are characterized by abnormal motility, where psychological or physiological stressors can notably affect motility response compared to ordinary people (23, 24). Moreover, mechanistic pathophysiology involving rectal desensitization to distention can also explain the symptom of FC (9). It should be noted that since FC is clinically different from constipation as an isolated symptom, a direct response to dietary fiber and lifestyle habits may not alleviate symptoms (9, 25).

While there was no specific examination of concomitant psychiatric disorders and social factors in this cohort, one of the current research purposes was to relate common gastrointestinal conditions among families with the prevalence of children's FGIDs. Interestingly, a higher incidence of functional dyspepsia (FD) was found in children with a positive family history of peptic ulcers. FD was the second most common prevalent FGIDs among participants of the current study, where 5% of the recruited samples fulfilled the Rome IV criteria of FD. These findings support a previous study result, where the incidence of FD in children was between 3% and 27% (26, 27). Altered visceral sensation, Helicobacter pylori infection, and gastrointestinal motor abnormalities have all been identified as major pathophysiological mechanisms for the development of FD (28, 29).

With regards to the prevalence of the most common GI disorders among families, it was found that belching, bloating, flatulence, IBS, acid reflux, heartburn, and GERD had the highest prevalence. Constipation was prevalent among 25% of families, which is considered a risk factor for their children. This was documented in a previous Saudi study as chronically constipated children were found to have a strong family history of constipation (11). However, there was no association between a family history of constipation and the prevalence of FC. On the contrary, there was a strong correlation between cesarean section and rates of FGIDs. The influence of the gut microbiota-brain axis on host physiology and the emergence of FGIDs may account for this connection. Colonization with bacteria comprising Bifidobacterium, Bacteroides, and Lactobacillus was found in neonates born *via* normal vaginal delivery. In contrast, early exposure to potentially harmful microbes such as Enterococcus, Clostridium, and Klebsiella was found in neonates born by Cesarean section (30, 31). Indeed, multivariate analysis confirmed the statistical significance between the development of different FGIDs and exposure to pathogenic bacteria (32). These results should be further explored to assess their possible relevance as the current study did not aim to investigate this specific association, and the impact of premature delivery and Caesarean section on FGIDs prevalence in neonates has not been thoroughly investigated. Therefore, we cannot neglect the similar results yielded by a study that focused on a limited time interval between neonates’ delivery and examination (21). It is possible that the impact of the birth delivery and date of the pregnancy initially exists but gradually fades as a result of various other variables, such as medication use, family dynamics, diet, environmental exposure, and infections, weakening its effect on the early years.

In the present report, it was found that there was a significant link between having FGIDs and developing GI discomfort following spicy foods and fizzy drink consumption. Although these food items have not been specifically studied, adverse food reactions are commonly claimed by populations with FGIDs (33–35). Whether these incompatibilities are a concomitant phenomenon or an underlying process in FGIDs is a crucial matter to be considered. Since this study was not explicitly designed to address this point, further studies are recommended to prove this causation.

It was evidenced from numerous perspectives, placebo-controlled clinical trials, and subsequent meta-analyses and systematic reviews that the usage of herbal medicinal products is considered a part of the routine treatment technique in many countries, including Saudi (19, 36–39). Therefore, there was an interest in exploring the use of herbal medicine and messages to alleviate any FGIDs symptoms in the current study. However, current results showed no significant use of herbal medicine to relieve or treat symptoms of FGIDs. This does not rule out the beneficial effects of complementary medicines on alleviating FGIDs symptoms (20). Future studies with a larger sample size to test this relationship are needed.

One of the best strengths of this study is using the ROME IV diagnostic questionnaire to identify specifically FGIDs based on self-reported symptoms; no physical assessments or invasive approaches are required to conduct this diagnosis. This was a great aid, mainly since this study was conducted during COVID-19 times when being in physical contact with children or their parents was not possible. Moreover, since the COVID-19 pandemic, most of the people living in Saudi Arabia, if not all of them, got experienced in using technology and families were required to include internet access in their homes to ease their children's access to school lessons every day. This bias is involved to all of the participants included. Nevertheless, determining. the incidence of FGIDs using the Rome IV DQ is considered a standard gold method in this field (9, 40). The tested translated DQ in this study is the first translated version available in Arabic- SA, which could provide researchers and clinicians in SA with a diagnostic tool for FGIDs. However, the outcomes of this research cannot be generalized to the targeted population of children, as it is a pilot study in this new field. The same researchers plan a more extensive study to use the current results and a larger calculated sample. This is to evaluate further the incidence of FGIDs in children 4+ years old in Jeddah and its countryside, Saudi Arabia.

## Data Availability

The raw data supporting the conclusions of this article will be made available by the authors, without undue reservation.

## References

[1] Corazziari E. Definition and epidemiology of functional gastrointestinal disorders. Best Pract Res Clin Gastroenterol. (2004) 18(4):613–31. 10.1016/j.bpg.2004.04.012 15324703

[2] Drossman DA. Functional gastrointestinal disorders: history, pathophysiology, clinical features and Rome IV. Gastroenterology. (2016) 150(6):1262–79.e2. doi 10.1053/j.gastro.2016.02.032 27144617

[3] Black CJ Drossman DA Talley NJ Ruddy J Ford AC. Functional gastrointestinal disorders: advances in understanding and management. Lancet. (2020) 396(10263):1664–74. doi 10.1016/S0140-6736(20)32115-2 33049221

[4] Drossman DA. The functional gastrointestinal disorders and the Rome III process. Gastroenterology. (2006) 130(5):1377–90. 10.1053/j.gastro.2006.03.008 16678553

[5] Benninga MA Nurko S Faure C Hyman PE st. James Roberts I Schechter NL. Childhood functional gastrointestinal disorders: neonate/toddler. Gastroenterology. (2016) 150(6):1443–55.e2. 10.1053/j.gastro.2016.02.016 27144631

[6] Zeevenhooven J Koppen IJN Benninga MA. The new Rome IV criteria for functional gastrointestinal disorders in infants and toddlers. Pediatr Gastroenterol Hepatol Nutr. (2017) 20(1):1–13. 10.5223/pghn.2017.20.1.1 28401050 PMC5385301

[7] Chey WD. The role of food in the functional gastrointestinal disorders: introduction to a manuscript series. Am J Gastroenterol. (2013) 108(5):694–7. 10.1038/ajg.2013.62 23545712

[8] Sperber AD Bangdiwala SI Drossman DA Ghoshal UC Simren M Tack J Worldwide prevalence and burden of functional gastrointestinal disorders, results of Rome foundation global study. Gastroenterology. (2021) 160(1):99–114.e3. 10.1053/j.gastro.2020.04.014 32294476

[9] Hyams JS di Lorenzo C Saps M Shulman RJ Staiano A van Tilburg M. Childhood functional gastrointestinal disorders: child/adolescent. Gastroenterology. (2016) 150(6):1456–68.e2. 10.1053/j.gastro.2016.02.015 PMC7104693 16678566

[10] Endo Y Shoji T Fukudo S. Epidemiology of irritable bowel syndrome. Ann Gastroenterol. (2015) 28(2):158–9. PMID: ; PMCID: 25830818 PMC4367204

[11] Hasosah M Alsahafi A Alghiribi A Alqarni N Babatin A Matrafi A Prevalence, characterization and risk factors of chronic constipation among Saudi children: a cross-sectional study. Int J Adv Res (Indore). (2018) 6(4):1319–24. 10.21474/IJAR01/6986

[12] Al-Mazrou YY Khan MU Aziz KM Farid SM. Factors associated with diarrhoea prevalence in Saudi Arabia. J Family Community Med. (1995) 2(1):27–34. 10.4103/2230-8229.98644 23012207 PMC3437149

[13] Ayoola EA. The clinical profile of cyclic vomiting syndrome in a regional hospital, Saudi Arabia. Trop Gastroenterol. [Internet] (2005) 26(3):126–8. Available from: http://www.ncbi.nlm.nih.gov/pubmed/16512460 16512460

[14] Vandenplas Y Alturaiki MA Al-Qabandi W AlRefaee F Bassil Z Eid B Middle East Consensus statement on the diagnosis and management of functional gastrointestinal disorders in &lt;12 months old infants. Pediatr Gastroenterol Hepatol Nutr. (2016) 19(3):153–61. 10.5223/pghn.2016.19.3.153 27738596 PMC5061656

[15] The Rome Foundation. Welcome to The Rome Foundation - Start Here. 2021 [cited 2022 Aug 2]. Available from: https://theromefoundation.org/#0

[16] Aljaaly E Khatib M. Evaluating the translatability process of the Rome IV diagnostic questionnaire to assess the prevalence of functional gastrointestinal disorders in Jeddah, Saudi Arabia. Curr Dev Nutr. (2021) 5(Suppl. 2):867–7. 10.1093/cdn/nzab048_002

[17] Blatch-Jones AJ Pek W Kirkpatrick E Ashton-Key M. Role of feasibility and pilot studies in randomised controlled trials: a crosssectional study. BMJ Open. (2018) 8(9):e022233. 10.1136/bmjopen-2018-022233 30257847 PMC6169762

[18] Cochran WG. Sampling techniques. 2nd ed. New York, NY: John Wiley and Sons, Inc. (1963).

[19] al Akeel MM al Ghamdi WM al Habib S Koshm M al Otaibi F. Herbal medicines: Saudi population knowledge, attitude, and practice at a glance. J Family Med Prim Care. (2017) 7(5):865–75. 10.4103/jfmpc.jfmpc_315_17 PMC6259532 30598925

[20] Holtmann G Schrenk D Madisch A Allescher HD Ulrich-Merzenich G Mearin F Use of evidence-based herbal medicines for patients with functional gastrointestinal disorders: a conceptional framework for risk-benefit assessment and regulatory approaches. Dig Dis. (2020) 38(4):269–79. 10.1159/000504570 31770769 PMC7384339

[21] Huang Y Tan SY Parikh P Buthmanaban V Rajindrajith S Benninga MA. Prevalence of functional gastrointestinal disorders in infants and young children in China. BMC Pediatr. (2021) 21(1):1–7. 10.1186/s12887-020-02457-3 33731059 PMC7968152

[22] Khayat A Algethami G Baik S Alhajori M Banjar D. The effect of using Rome IV criteria on the prevalence of functional abdominal pain disorders and functional constipation among children of the western region of Saudi Arabia. Glob Pediatr Health. (2021) 8:2333794X211022265. 10.1177/2333794X211022265 34104704 PMC8170292

[23] Saito YA Locke GR Weaver AL Zinsmeister AR Talley NJ. Diet and functional gastrointestinal disorders: a population-based case-control study. Am J Gastroenterol. (2005) 100(12):2743–8. 10.1111/j.1572-0241.2005.00288.x 16393229

[24] Parkman HP Hasler WL Fisher RS, American Gastroenterological Association. American Gastroenterological Association technical review on the diagnosis and treatment of gastroparesis. Gastroenterology. (2004) 127(5):1592–622. 10.1053/j.gastro.2004.09.055 15521026

[25] Piccoli de Mello P Eifer DA Daniel de Mello E. Use of fibers in childhood constipation treatment: systematic review with meta-analysis. J Pediatr (Rio J). (2018) 94(5):460–70. 10.1016/j.jped.2017.10.014 29474804

[26] Alonso-Bermejo C Barrio J Fernández B García-Ochoa E Santos A Herreros M Functional gastrointestinal disorders frequency by Rome IV criteria. An Pediatr (Engl Ed). (2022) 96(5):441–7. 10.1016/j.anpedi.2021.05.021 35534416

[27] Romano C Valenti S Cardile S Benninga MA. Functional dyspepsia: an enigma in a conundrum. J Pediatr Gastroenterol Nutr. (2016) 63(6):579–84. 10.1097/MPG.0000000000001344 27437927

[28] Thumshirn M. Pathophysiology of functional dyspepsia. Gut. (2002) 51(Supplement 1):i63–6. 10.1136/gut.51.suppl_1.i63 12077069 PMC1867724

[29] Shava U Srivastava A Mathias A Kumar N Yachha SK Gambhir S Functional dyspepsia in children: a study of pathophysiological factors. J Gastroenterol Hepatol. (2021) 36(3):680–6. 10.1111/jgh.15193 32710649

[30] Montoya-Williams D Lemas DJ Spiryda L Patel K Carney OO Neu J The neonatal microbiome and its partial role in mediating the association between birth by cesarean section and adverse pediatric outcomes. Neonatology. (2018) 114(2):103–11. 10.1159/000487102 29788027 PMC6532636

[31] Velasco-Benitez CA Axelrod CH Gutierrez S Saps M. The relationship between prematurity, method of delivery, and functional gastrointestinal disorders in children. J Pediatr Gastroenterol Nutr. (2020) 70(2):e37–40. 10.1097/MPG.0000000000002543 31978026

[32] Shin A Preidis GA Shulman R Kashyap PC. The gut microbiome in adult and pediatric functional gastrointestinal disorders. Clin Gastroenterol Hepatol. (2019) 17(2):256–74. 10.1016/j.cgh.2018.08.054 30153517 PMC6314902

[33] Eswaran S Tack J Chey WD. Food: the forgotten factor in the irritable bowel syndrome. Gastroenterol Clin North Am. (2011) 40(1):141–62. 10.1016/j.gtc.2010.12.012 21333905

[34] Wilson K Hill RJ. The role of food intolerance in functional gastrointestinal disorders in children. Aust Fam Physician. (2014) 43(10):686–9. PMID: 25286424

[35] Wilder-Smith CH Materna A Wermelinger C Schuler J. Fructose and lactose intolerance and malabsorption testing: the relationship with symptoms in functional gastrointestinal disorders. Aliment Pharmacol Ther. (2013) 37(11):1074–83. 10.1111/apt.12306 23574302 PMC3672687

[36] Rich G Shah A Koloski N Funk P Stracke B Köhler S A randomized placebo-controlled trial on the effects of Menthacarin, a proprietary peppermint- and caraway-oil-preparation, on symptoms and quality of life in patients with functional dyspepsia. Neurogastroenterol Motil. (2017) 29(11):e13132. 10.1111/nmo.13132 28695660

[37] von Arnim U Peitz U Vinson B Gundermann KJ Malfertheiner P. STW 5, A phytopharmacon for patients with functional dyspepsia: results of a multicenter, placebo-controlled double-blind study. Am J Gastroenterol. (2007) 102(6):1268–75. 10.1111/j.1572-0241.2006.01183.x 17531013

[38] Madisch A Holtmann G Mayr G Vinson B Hotz J. Treatment of functional dyspepsia with a herbal preparation. A double-blind, randomized, placebo-controlled, multicenter trial. Digestion. (2004) 69(1):45–52. 10.1159/000076546 14755152

[39] Holtmann G Talley NJ. Herbal medicines for the treatment of functional and inflammatory bowel disorders. Clin Gastroenterol Hepatol. (2015) 13(3):422–32. 10.1016/j.cgh.2014.03.014 24674944

[40] Rasquin A di Lorenzo C Forbes D Guiraldes E Hyams JS Staiano A Childhood functional gastrointestinal disorders: child/adolescent. Gastroenterology. (2006) 130(5):1527–37. 10.1053/j.gastro.2005.08.063 16678566 PMC7104693

